# Three Complete Mitochondrial Genomes of *Orestes guangxiensis*, *Peruphasma schultei*, and *Phryganistria guangxiensis* (Insecta: Phasmatodea) and Their Phylogeny

**DOI:** 10.3390/insects12090779

**Published:** 2021-08-31

**Authors:** Ke-Ke Xu, Qing-Ping Chen, Sam Pedro Galilee Ayivi, Jia-Yin Guan, Kenneth B. Storey, Dan-Na Yu, Jia-Yong Zhang

**Affiliations:** 1 College of Chemistry and Life Science, Zhejiang Normal University, Jinhua 321004, China; xkk20190815@163.com (K.-K.X.); 15157932450@163.com (Q.-P.C.); paydrov17@gmail.com (S.P.G.A.); a1345413239@163.com (J.-Y.G.); ydn@zjnu.cn (D.-N.Y.); 2 Department of Biology, Carleton University, Ottawa, ON K1S 5B6, Canada; KennethStorey@cunet.carleton.ca; 3 Key Lab of Wildlife Biotechnology, Conservation and Utilization of Zhejiang Province, Zhejiang Normal University, Jinhua 321004, China

**Keywords:** stick insects, phylogenetic relationships, protein-coding gene, monophyly

## Abstract

**Simple Summary:**

Twenty-seven complete mitochondrial genomes of Phasmatodea have been published in the NCBI. To shed light on the intra-ordinal and inter-ordinal relationships among Phasmatodea, more mitochondrial genomes of stick insects are used to explore mitogenome structures and clarify the disputes regarding the phylogenetic relationships among Phasmatodea. We sequence and annotate the first acquired complete mitochondrial genome from the family Pseudophasmatidae (*Peruphasma schultei*), the first reported mitochondrial genome from the genus *Phryganistria* of Phasmatidae (*P. guangxiensis*), and the complete mitochondrial genome of *Orestes guangxiensis* belonging to the family Heteropterygidae. We analyze the gene composition and the structure of the three mitochondrial genomes. We recover the monophyly of Phasmatodea and show the sister-group relationship between Phasmatodea and Mantophasmatodea after removal of the Embioptera and Zoraptera species. We recover the monophyly of Heteropterygidae and the paraphyly of Diapheromeridae, Phasmatidae, Lonchodidae, Lonchodinae, and Clitumninae.

**Abstract:**

Insects of the order Phasmatodea are mainly distributed in the tropics and subtropics and are best known for their remarkable camouflage as plants. In this study, we sequenced three complete mitochondrial genomes from three different families: *Orestes guangxiensis, Peruphasma schultei,* and *Phryganistria guangxiensis*. The lengths of the three mitochondrial genomes were 15,896 bp, 16,869 bp, and 17,005 bp, respectively, and the gene composition and structure of the three stick insects were identical to those of the most recent common ancestor of insects. The phylogenetic relationships among stick insects have been chaotic for a long time. In order to discuss the intra- and inter-ordinal relationship of Phasmatodea, we used the 13 protein-coding genes (PCGs) of 85 species for maximum likelihood (ML) and Bayesian inference (BI) analyses. Results showed that the internal topological structure of Phasmatodea had a few differences in both ML and BI trees and long-branch attraction (LBA) appeared between Embioptera and Zoraptera, which led to a non-monophyletic Phasmatodea. Consequently, after removal of the Embioptera and Zoraptera species, we re-performed ML and BI analyses with the remaining 81 species, which showed identical topology except for the position of *Tectarchus ovobessus* (Phasmatodea). We recovered the monophyly of Phasmatodea and the sister-group relationship between Phasmatodea and Mantophasmatodea. Our analyses also recovered the monophyly of Heteropterygidae and the paraphyly of Diapheromeridae, Phasmatidae, Lonchodidae, Lonchodinae, and Clitumninae. In this study, *Peruphasma schultei* (Pseudophasmatidae), *Phraortes* sp. YW-2014 (Lonchodidae), and species of Diapheromeridae clustered into the clade of Phasmatidae. Within Heteropterygidae, *O. guangxiensis* was the sister clade to *O. mouhotii* belonging to Dataminae, and the relationship of (Heteropteryginae + (Dataminae + Obriminae)) was recovered.

## 1. Introduction

Stick and leaf insects (Phasmatodea) belong to an order of polyneopteran insects, which includes over 3000 recognized species subdivided into approximately 500 genera, distributed across major landmasses [1]. Phasmatodea are the longest insects among extant species, mainly distributed in tropical and subtropical regions, with a few species in temperate regions [2]. Phasmatodea are herbivorous insects and have a strong ability to masquerade as bark, leaves, and twigs, which provide them with camouflage [3,4,5]. Consequently, it is difficult to explore the phylogenetic relationship of Phasmatodea by the degree of convergence of morphology [6].

The phylogenetic relationship of Phasmatodea within Polyneoptera has been under debate. Polyneoptera includes ten insect orders of Blattodea, Dermaptera, Embioptera, Grylloblattodea, Mantodea, Mantophasmatodea, Orthoptera, Phasmatodea, Plecoptera, and Zoraptera. Both morphological and molecular data highly support Embioptera as the sister group of Phasmatodea [7,8,9,10,11,12]. Some studies showed that Phasmatodea formed a sister group to the Mantophasmatodea [13,14,15,16], whereas other analyses alternately supported a closer relationship between Phasmatodea and Orthoptera [8,17,18,19]. Moreover, recent research based on mitochondrial genome data supported the idea that Phasmatodea had a close relationship with Embioptera and Zoraptera [20,21,22].

The high-level phylogenetic relationships of Phasmatodea are currently not sufficiently clear [23,24]. Several authors have suggested that Phasmatodea should be divided into two suborders: Timematodea and Euphasmatodea (Verophasmatodea) [1,5,25,26]. The Euphasmatodea includes thirteen families: Aschiphasmatidae, Damasippoididae, Prisopodidae, Anisacanthidae, Bacillidae, Heteropterygidae, Phylliidae, Agathemeridae, Heteronemiidae, Pseudophasmatidae, Diapheromeridae, Lonchodidae, and Phasmatidae [2]. However, the wingless Nearctic walking-stick, *Timema* of family Timematidae, is the only genus within the one family in Timematodea [27]. Neverthless, Simon et al. demonstrated a basal dichotomy of Aschiphasmatodea and the Neophasmatodea in Euphasmatodea [28]. This result was also supported by Tihelka et al. [29]. The monophyly of Phasmatodea remains in dispute because of the phylogenetic position of genus *Timema*. Most studies based on morphology, transcriptome, and mitochondrial genome data have shown that the monophyly of Phasmatodea can be confirmed, and *Timema* is recognized as the sister group to all remaining phasmids [28,30,31,32,33]. Nevertheless, data on mitochondrial genomes considered that *Timema* did not belong to the Euphasmatodea, but grouped with other orders (e.g., Orthoptera and Embioptera) [6,34]. This result coincided with the study about the morphology of *Timema* specie*s* in egg that indicated that *Timema* was a separate lineage [35]. Based on 16S, 12S RNA genes, and protein codon genes, Song et al. [22] constructed nine phylogenetic trees, and eight of these failed to recover the monophyly of Phasmatodea because the clade of Embioptera and Zoraptera that existed in long-branch attraction (LBA) was a sister group to Euphasmatodea, whereas only one phylogenetic tree supported the monophyly of Phasmatodea, as found in Song et al. [36]. In many phylogenetic analyses, especially those based on mitochondrial genomes, biases associated with LBA have been found [37].

Insect mitochondrial genomes normally have 37 genes (thirteen protein-coding genes, two ribosomal RNAs, and 22 transfer RNA genes) and a control region (CR) and are usually a 14–20 kb double-stranded circular molecule [38]. The mitochondrial genome has been widely used for phylogenetic analyses due to its simple and stable gene organization, lack of genetic recombination, and fast evolution rate, etc. [21,39,40,41]. Many researchers have discussed the phylogenetic relationships among insect orders using mitochondrial genomes, such as Diptera [42], Orthoptera [43], Hymenoptera [44], and Coleoptera [45]. Hence, the mitochondrial genome of stick insects was used for phylogenetic analysis in this study.

At present, 27 complete mitochondrial genomes of Phasmatodea have been published in the NCBI. To shed light on the intra-ordinal and inter-ordinal relationships among Phasmatodea, we sequenced and annotated three complete mitochondrial genomes from *Orestes guangxiensis* (Bi & Li, 1994) (Heteropterygidae), *Phryganistria guangxiensis* Chen & He, 2008 (Phasmatidae), and *Peruphasma schultei* Conle & Hennemann, 2005 (Pseudophasmatidae). This included the first acquired complete mitochondrial genome from the Pseudophasmatidae and the first reported mitochondrial genome of *Phryganistria* (Phasmatidae). Moreover, we analyzed the gene composition and the structure of the three mitochondrial genomes.

## 2. Materials and Methods

### 2.1. Sampling Collection and DNA Extraction

*Orestes guangxiensis* and *Ph*. *guangxiensis* were collected from Jinxiu, Guangxi province, China, whereas *Pe. schultei* was retrieved from an insect pet market in China, the source area being Northern Peru. According to their morphological characters, these specimens were identified by JY Zhang and stored at −40℃ in the Zhang laboratory, College of Life Sciences and Chemistry, Zhejiang Normal University, China. Total DNA was extracted from a piece of foreleg muscle using a Universal Genomic DNA Kit (Co Win Biosciences Company, Beijing, China).

### 2.2. PCR Amplification and Sequencing

The DNA from each of the three species was amplified using eight pairs of universal primers, as described in Zhang et al. [46], but there were still some vacancies. We then used Primer Premier 5.0 to design species-specific primers based on known sequences from universal primers [47] (Table S1) and used normal PCR (product length <3000 bp) as well as long PCR (product length >3000 bp) methods for amplification [46]. Takara *Taq* polymerase and Takara *LATaq* DNA polymerase were used, respectively (Takara, Dalian, China), in a 50 µL reaction volume. Reaction systems and cycling conditions for normal PCR and long PCR were as described in Zhang et al. [46]. All PCR products were sequenced in both directions using the primer-walking method and ABI3730XL by Sangon Biotech Company (Shanghai, China).

### 2.3. Mitochondrial Genome Annotation and Sequence Analyses

The fragments obtained by Sanger dideoxy sequencing were assembled with DNASTAR Package v.7.1 [48]. The tRNA genes were identified using the MITOS web server (http://mitos.bioinf.uni-leipzig.de/index.py (accessed on 15 July 2021)) [49]. Based on the homologous sequences of mitochondrial genomes from other stick insect species, we used Clustal X [50] to determine the two rRNA genes (12S and 16S rRNA). The remaining 13 protein-coding genes were analyzed using Mega 7.0 [51] to translate amino acids using the invertebrate mitochondrial genetic code and find open reading frames [52]. The AT content, codon usage, and relative synonymous codon usage (RSCU) of protein-coding genes were calculated by PhyloSuite 1.2.2 [53]. GC and AT skews were calculated according to the formula: AT skew = (A − T)/(A + T), GC skew = (G − C)/(G + C) [54].

### 2.4. Phylogenetic Analyses

To illuminate the phylogenetic relationships of Phasmatodea, we first performed maximum likelihood (ML) and Bayesian inference (BI) analyses based on data from 85 species, including the three newly determined sequences, sixty-five previously sequenced mitochondrial genomes, and 13 PCGs of seventeen species of Phasmatodea assembled from transcriptome data [14,33,34,36,55,56,57,58,59,60,61,62,63,64,65,66,67,68,69,70,71,72,73,74,75,76,77] (Table 1, Table 2 and Table S2). Long-branch attraction between Embioptera and Zoraptera appeared in both ML and BI phylogenetic trees and affected the stability of the topology. This phenomenon probably occurred because of phylogenetic artifacts generated by the mitochondrial genome [78]. For LBA, the following measures are recommended: increasing samples [79]; removing long-branch groups [80]; or removing sites with rapid evolutionary rates [81]. Adding sequences was not feasible because all current mitochondrial genome sequences of Embioptera and Zoraptera were used in this study. Then, we attempted to remove sites with fast evolutionary rates, but nevertheless, the results were not very reliable. Therefore, we reconstructed BI and ML phylogenetic trees with 13 concatenated PCG sequences of 81 species after removing representative species of Embioptera and Zoraptera. Three species of Archaeognatha, *Nesomachilis australica*, *Pedetontus silvestrii*, and *Trigoniophthalmus alternatus* were used as outgroups (Table 2). We aligned each of the 13 protein-coding genes using Clustal W in the program Mega 7.0 [51] and used the program Gblock 0.91b to identify conserved regions [82]. The resulting alignments were concatenated with Geneious 8.1.6 [83]. Because the third codon positions had saturated by DAMBE 4.2.13 [84], we used the Bayesian inference (BI) and maximum likelihood (ML) methods with the first and second codon datasets to analyze the phylogenetic relationships. ML analysis was implemented by IQ-TREE v.2.1.2 with the best model GTR + I + G that was acquired by ModelFinder [85,86]. BI analysis was carried out by MrBayes 3.2 with GTR + I + G [87] and was set for 10 million generations with sampling every 1000 generations, and the first 25% of generations were discarded as burn-in.

## 3. Results and Discussion

### 3.1. Mitochondrial Genome Organization and Composition

**Figure 1:**
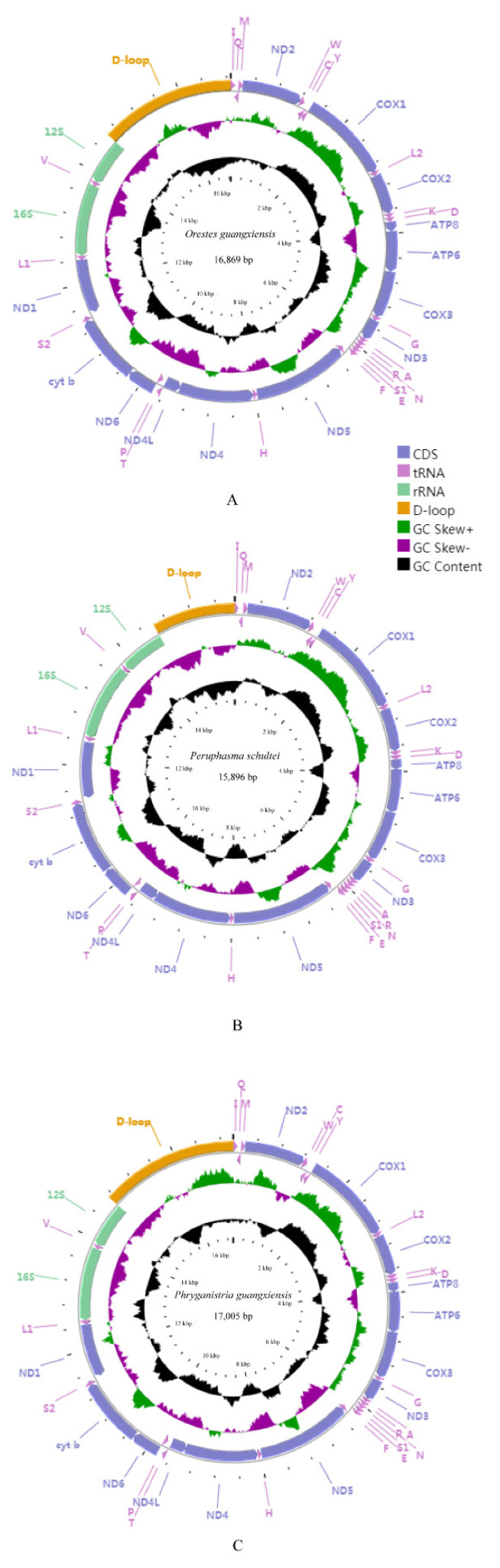
Mitochondrial genome maps of *O. guangxiensis* (**A**), *Pe. schultei* (**B**), and *Ph. guangxiensis* (**C**). The first circle shows the gene map (PCGs, rRNAs, tRNAs, and the AT-rich region). The genes shown outside the map are coded on the majority strand (J-strand), whereas the genes inside the map are coded on the minority strand (N-strand). The second circle shows the GC skew and the third shows the GC content. GC content and GC skew are plotted as the deviation from the average value of the entire sequence.

The lengths of the three complete mitochondrial genomes of *O. guangxiensis, Pe. schultei,* and *Ph. guangxiensis* were 16,869 bp, 15,896 bp, and 17,005 bp, respectively (Figure 1). All three genomes were deposited in GenBank, with accession numbers MW450873, MW450874, and MW450875, respectively. Mitochondrial genomes of the three species had the same genes and gene order as those of other published stick insects, which have 37 genes, including 13 PCGs, 22 tRNA genes, and two rRNA genes. Currently, the gene arrangement of published stick insects is similar to the assumed common ancestor of insects, except for *Ramulus hainanense* (CR-trnM-trnQ-trnI-CR-trnI-trnQ-trnM) and *Megalophasma granulatum* (trnR-trnA) [6,14,15,22,30,89,90,91]. According to the previously published complete mitochondrial genomes of stick and leaf insects, we found that the differing lengths of the Phasmatodea genomes (15,590–18,248 bp) were caused mainly by the size of the A + T-rich region, gene overlaps, and different intergenic nucleotides (IGNs). The sequence length of *Pe. schultei* (15,896 bp), with a short A + T-rich region (<1500 bp), was the shortest after that of *R. hainanense* (15,590 bp). The three species have short intergenic regions ranging from 1 to 18 bp. The whole mitochondrial genome of *Ph. guangxiensis,* which contained additional IGNs (136 bp), was longer than that of *O. guangxiensis* (Tables S3–S5). The nucleotide composition of the *O. guangxiensis*, *Pe. schultei,* and *Ph. guangxiensis* mitochondrial genomes had a high A + T bias of 75.6%, 76.6%, and 76.8%, respectively. All three species showed a positive AT skew and negative GC skew (Table 3). The content of A was more than T, and the content of C was higher than G, which also occurred in the sequences of previously studied stick insects (Table S6) [6,14,15,22,30,89,90,91].

### 3.2. Protein-Coding Genes and Codon Usages

The locations of the 13 PCGs within the three mitochondrial genomes were identical to those of most stick insects (Tables S3–S5). Four PCGs (ND1, ND4, ND4L, and ND5) were located in the minority strand (N-strand), whereas the remaining PCGs were coded on the majority strand (J-strand). The total lengths of the 13 protein-coding genes (PCGs) in *O. guangxiensis*, *Pe. schultei,* and *Ph. guangxiensis* were 11,112 bp, 11,100 bp, and 11,121 bp, respectively (Table 3). Among the three mitochondrial genomes, all PCG initiation codons used ATN (N represents A, G, C, or T), except for ND4L of *Pe. schultei,* which started with GTG. GTG as a start codon has also been found in ND4 of *Megalophasma granulatum* [91]. ATN is an accepted canonical initiation codon for insect mitochondrial genomes [92]. Of the stick insects that used ATN as an initiation codon, most used ATA, ATG, and ATT, with only a few using ATC [6,14,15,22,30,89,90,91]; only ATP8 (*Pe. schultei*) used the ATC start codon in this study. The typical termination codons (TAA/TAG) were found in most PCGs, except for some incomplete terminal codons, such as T used for COX2 (*O. guangxiensis*, *Pe. schultei,* and *Ph. guangxiensis*), ND1 (*Ph. guangxiensis*), ND3 (*O. guangxiensis* and *Pe. schultei*), ND4L (*Pe. schultei*), and ND5 (*O. guangxiensis* and *Pe. schultei*). Incomplete termination codons have also been found in other insects [65,93,94,95,96]. Incomplete stop codons play a significant role in polycistronic transcription cleavage and polyadenylation processes [97]. High A + T bias was also found in the PCGs of *O. guangxiensis*, *Pe. schultei,* and *Ph. guangxiensis,* which were 74.2%, 75.5%, and 75.8%, respectively. The PCGs of the majority strand displayed positive AT skews and negative GC skews, whereas the minority strand displayed negative AT skews and positive GC skews. The A skew (the content of A > T) and C skew (the content of C > G) of the minority strand was greater than on the majority strand (Table 3).

**Figure 2:**
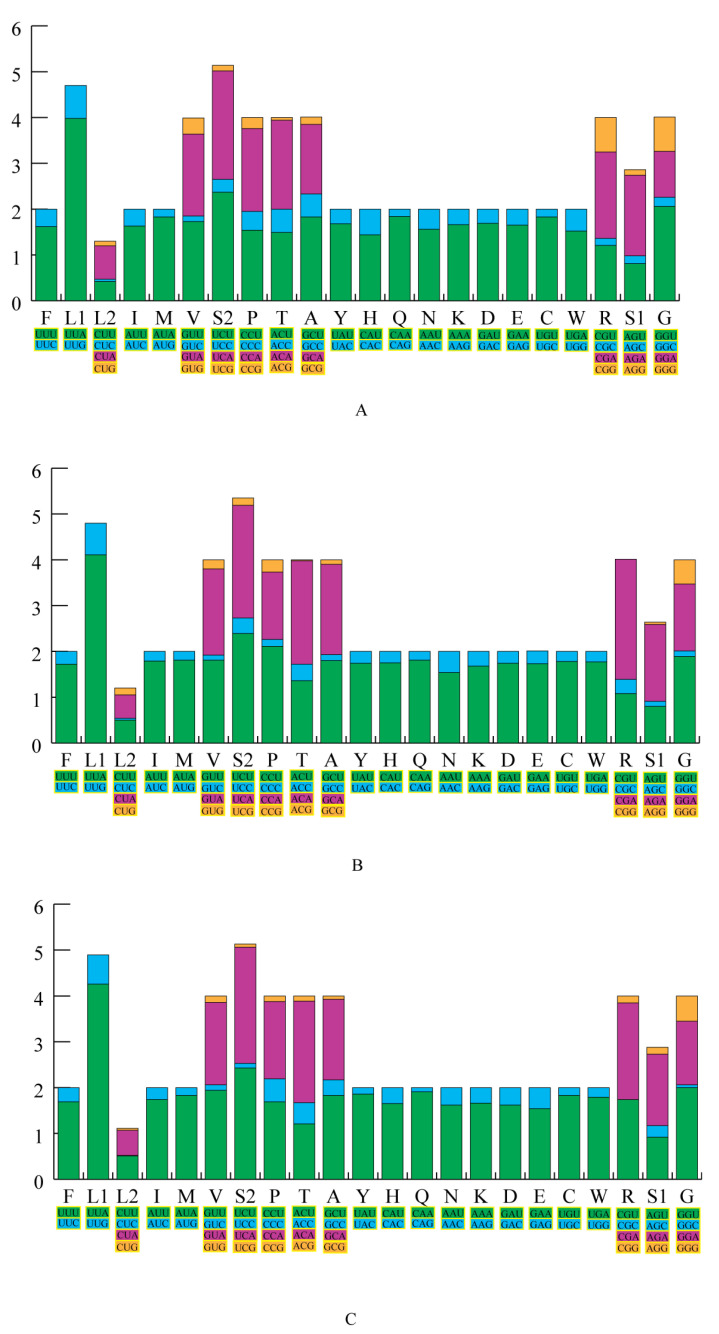
The relative synonymous codon usage (RSCU) in three Phasmatodea mitochondrial genomes. The RSCU of the mitochondrial genome in *O. guangxiensis* (**A**), *Pe. schultei* (**B**), and *Ph. guangxiensis* (**C**).

We calculated the relative synonymous codon usage (RSCU) of the three mitochondrial genomes (Figure 2, Table S7). The results showed that A or T nucleotides were used in high frequency in the third codon position compared to other nucleotides, and that A was used more often than T. The most frequent codons used were UUU (Phe), UUA (Leu), AUU (Ile), and AUA (Met) and were used >280 times in the PCGs of *O. guangxiensis*, *Pe. schultei,* and *Ph. guangxiensis* mitochondrial genomes. In contrast, codons with a third codon G or C were used very rarely (≤10), such as CUC (Leu), UGC (Cys), CGC (Arg), GCG (Ala) (≤5), etc. This may be a kind of AT mutation bias that has an obvious influence on codon usage [98,99].

### 3.3. Ribosomal RNAs and Transfer RNAs

The mitochondrial genomes of *O. guangxiensis*, *Pe. schultei,* and *Ph. guangxiensis* each had 22 tRNA genes, as in other Phasmatodea mitogenomes [6,14,15,22,30,89,90,91]. The total tRNA sizes of *O. guangxiensis*, *Pe. schultei,* and *Ph. guangxiensis* were 1463 bp, 1432 bp, and 1468 bp, respectively, with a high A + T bias of 78.3%, 77.6%, and 79.2%. Among the 22 tRNA genes of the three species, most secondary structures of the tRNA genes can fold into the common cloverleaf model, except for trnS1 (*O. guangxiensis*), which lacks the dihydrouridine (DHC) arm, and trnN (*O. guangxiensis* and *Pe. schultei*) and trnP (*O. guangxiensis*), which had lost the TΨC loops (Figures S1–S3). A lack of DHC arms or TΨC loops exists in other stick insects and various insects in general [62,91,100,101,102], and these have lower translational activity compared to the normal structures [103]. We also found a few mismatched pairs, such as unmatched A-A base pairs in trnS1 of the three stick species, A-G in trnW (*O. guangxiensis* and *Pe. schultei*), C-A in trnG (*Ph. guangxiensis*), U-U in trnV of the three stick insects, as well as trnA (*O. guangxiensis* and *Pe. schultei*), trnY (*Ph. guangxiensis*), trnS2, and trnL1(*Pe. schultei*). Mismatched pairs may affect aminoacylation and translation [104].

The mitochondrial genomes of these three stick insects, similar to other species in Phasmatodea, contained two rRNAs genes [6,15]. The 16S rRNA gene in *O. guangxiensis*, *Pe. schultei,* and *Ph. guangxiensis* was located between trnL1 and trnV, with a length of 1291 bp, 1281 bp, and 1328 bp, respectively, whereas the 12S rRNA was located between trnV and the CR, with sizes of 788 bp, 774 bp, and 796 bp, respectively. The AT content of the two rRNAs in *O. guangxiensis* (78.3%), *Pe. schultei* (77.7%), and *Ph. guangxiensis* (77.9%) were each higher than the average AT content of the 13 PCGs (Table 3). We found that the AT skew values of the two rRNAs in *O. guangxiensis*, *Pe. schultei,* and *Ph. guangxiensis* were 0.25, 0.22, and 0.20, respectively. Meanwhile, the GC skew was highly negative, with values around 0.3 (Table 3).

### 3.4. A + T-Rich Region

The large non-coding region of *O. guangxiensis*, *Pe. schultei,* and *Ph. guangxiensis* between trnI and 12S rRNA was an A + T-rich region with lengths of 2238 bp, 1294 bp, and 2286 bp, respectively (Table 3). Compared with mitogenomes from other Phasmatodea, the length of the A + T-rich region in *Pe. schultei* was the shortest, except for *Ramulus hainanense* (774 bp) (FJ156750). According to the published complete Phasmatodea mitochondrial genomes, the longest A + T-rich region was found in *Cryptophyllium tibetense* (3701 bp) (KX091862). In the mitochondrial genomes of *O. guangxiensis*, *Pe. schultei,* and *Ph. guangxiensis,* the content of A + T in the control regions was 79.6%, 82.5%, and 79.1%, respectively, which was higher than other partitions of mitochondrial genomes. The A + T-rich regions of the three species each showed positive AT skew values and negative GC skew values (Table 3). The A + T region embodied the origin sites and essential regulatory elements needed for transcription and replication [105,106,107].

**Figure 3:**
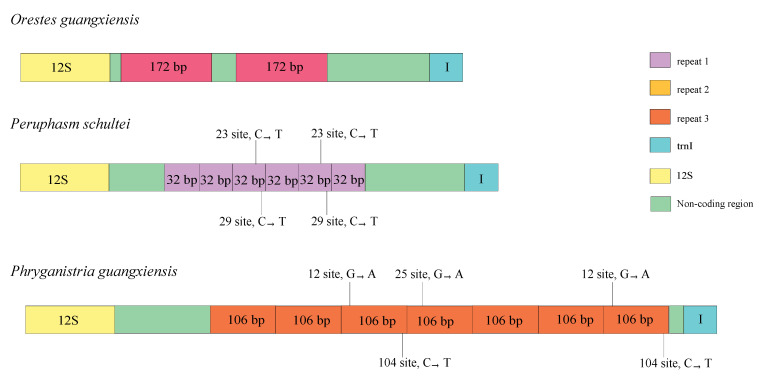
Organizations of the repeat regions in the control region of *O. guangxiensis*, *Pe. schultei*, and *Ph. guangxiensis*.

Repeat regions were observed in *O. guangxiensis*, *Pe. schultei,* and *Ph. guangxiensis* (Figure 3). The A + T-rich region of *Ph. guangxiensis* possessed seven copies of tandem repeats regions with a length of 106 bp, whereas the A + T-rich region of *Pe. schultei* contained six tandem repeat regions of a 32 bp sequence. However, two repeats (172 bp) in *O. guangxiensis* were not tandem (Figure 3). Tandem repeats in the A + T rich region have also been observed in many Phasmatodea species. In the research of Kômoto et al., the presence of tandem repeats in the A + T region was also detected in eight Phasmatodea species, such as *Entoria nuda* (ten tandem repeats of a 128–129 bp sequence) and *Ramulus mikado* (two tandem repeats of a 149 bp sequence and seven tandem repeats of 125 bp) [6]. Two identical copies of a 64 bp tandem repeat were discovered in *Ramulus hainanense* (FJ15676), and *Eurycantha calcarata* included twenty-two tandem repeats of a 32 bp fragment (MW915467). Twenty-two tandem repeats of a 21 bp sequence were found in *Cryptophyllium tibetense* (KX091862), and *Extatosoma tiaratum* possessed three tandem repeats of a 128-129 bp fragment [30]. *Phraortes* sp. 1 NS-2020 contained two tandem repeats with lengths of 20 bp [22]. The secondary structure of these repeat units was predicted by RNAalifold in 15 species [108,109,110]. We found that most of these tandem repeats in the A + T region could form the stem–loop structure (Figure S4). Repeat regions of the mitochondrial A + T region also existed in other insects. The A + T-rich region of *Theopompa* sp.YN (Mantodea: Mantidae) contained three tandem repeats of a 200 bp sequence [96]. Two tandem repeats of a 90 bp sequence, three tandem repeats of a 100 bp sequence, and six tandem repeats of a 50 bp sequence were observed in *Serratella zapekinae* (Ephemeroptera: Ephemerellidae) [63]. The cause of tandem repeats may be slipped-strand mispairing in mitochondrial genome replication [111].

### 3.5. Intergenic and Overlap Regions

The mitochondrial genomes of Phasmatodea, including the three stick insects in this study, were compact, with intergenic regions usually not exceeding 20 bp [6,14,15,22,30,89,90,91]. The mitochondrial genomes of *O. guangxiensis*, *Pe. schultei,* and *Ph. guangxiensis* contained 7, 5, and 12 intergenic regions with total lengths of 17 bp, 41 bp, and 40 bp, respectively. We observed the longest intergenic spacer between ND1 and trnL1 and the second-longest between trnI and trnQ within the mitochondrial genomes of *Pe. schultei*, with lengths of 18 bp and 13 bp, respectively. Overall, insertions between genes ranged from 1 to 8 residues in the three stick insects (Tables S3–S5).

The mitochondrial genomes of *O. guangxiensis*, *Pe. schultei,* and *Ph. guangxiensis* had 14, 10, and 13 overlaps with a total length of 39 bp, 28 bp, and 38 bp, respectively. Coincidentally, we observed that the three phasmatodean species shared four pairs of gene overlaps: trnW/trnC (8 bp), COX1/trnL2 (5 bp), ATP8/ATP6 (4 bp), and ATP6/COX3 (1 bp). The overlapping between ATP6 and COX3 was an A that also exists in all published Phasmatodea mitochondrial genomes [6,14,15,22,30,89,90,91]. An 8 bp (AAGYCTTA) overlap was also found between trnW and trnC that is present in all published sequences except *Timema californicum* and *Dryococelus australis*. Simultaneously, a pentanucleotide TCTAA consensus motif existed in the overlap regions situated between COX1 and trnL2 of a few other stick insects [15,91].

### 3.6. Phylogenetic Analyses

When we analyzed the phylogenetic relationship using the 13PCGs of 85 species, including Embioptera and Zoraptera, the Bayesian tree was different from the maximum likelihood inference tree, mainly in the internal topological structure of Phasmatodea (Figures S5 and S6). We found that Phasmatodea was paraphyletic because the clade of Zoraptera and Embioptera clustered into the Phasmatodea, as also reported by Song et al. based on mitochondrial genome sequence data [22,36]. Zoraptera was the sister clade to Embioptera, caused by long-branch attraction, as found in Ma et al. [20].

**Figure 4:**
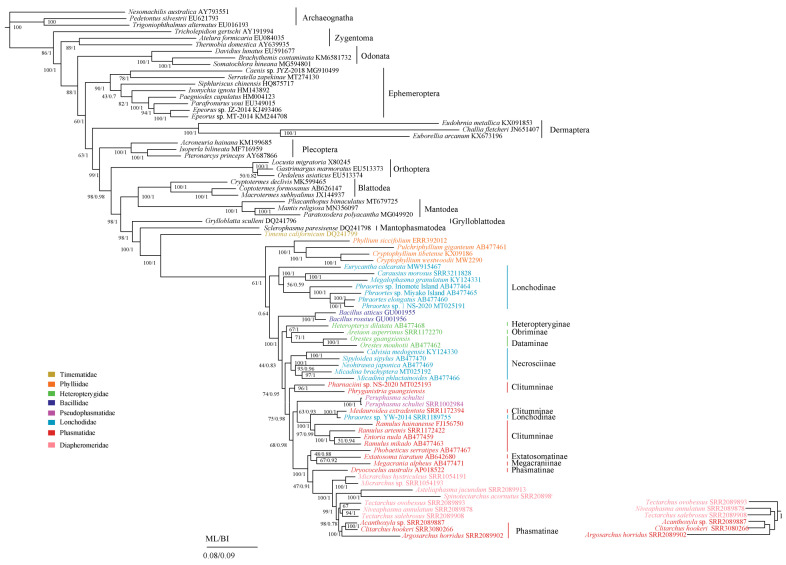
Phylogenetic relationships of Phasmatodea among 85 species including *O. guangxiensis*, *Pe. schultei,* and *Ph. guangxiensis*, inferred from ML and BI analyses based on the nucleotide dataset of the 13 mitochondrial protein-coding genes. Three species of Archaeognatha were used as outgroups. The GenBank and SRA accession numbers of all species are shown in the figure. Numbers around the nodes are the posterior probabilities of BI (right) and the bootstrap values of ML (left). Different colors in the species names correspond to the family names.

After removal of the Embioptera and Zoraptera species, we re-performed ML and BI analyses with the remaining 81 species, which showed identical topology except for the position of *Tectarchus ovobessus* (Phasmatodea). Figure 4 shows that Odonata was the basal group of Pterygota, and Ephemeroptera was a sister clade to the Polyneoptera, as also reported by some other molecular and morphological studies [62,112,113]. The monophyly of Polyneoptera also was supported. We recovered the monophyly of Phasmatodea, and the sister-group relationship between Phasmatodea and Mantophasmatodea was supported by current phylogenetic analyses after removing Zoraptera and Embioptera. Phasmatodea was divided into two branches: Timematodea and Euphasmatodea (Figure 4).

At the family level, our data supported the monophyly of Heteropterygidae but Diapheromeridae, Phasmatidae, and Lonchodidae were not recovered. Lonchodidae consists of two subfamilies, Lonchodinae and Necrosciinae, but did not form a clade, which was also reported by Forni et al., Kômoto et al., and Song et al. [6,22,33]. However, the phylogenetic relationship among Dataminae, Heteropteryginae, and Obriminae of Heteropteridae is still controversial. In this study, *O. guangxiensis* was the sister clade to *O. mouhotii* belonging to Dataminae, and Heteropteryginae was the sister clade to (Dataminae + Obriminae). Meanwhile, the same conclusion was also obtained by some research that utilized morphological data [35,114] and molecular data [115]. By contrast, Bank et al., based on three nuclear data sets (18S, 28S and H3) and four mitochondrial data genes (COX1, COX2, 12S, and 16S), were in favor of the clade of Dataminae + (Heteropteryginae + Obriminae) [116], consistent with previous results [117,118,119]. By contrast, other studies hypothesized that Obriminae and the clade of (Heteropteryginae + Dataminae) had a close phylogenetic relationship [5,28,120,121]. The Pseudophasmatidae (*Pe. schultei*) should be a separate clade, but our molecular phylogenetic trees showed that it was classified into Phasmatidae, and, at the same time, *Phraortes* sp. YW-2014 (Lonchodidae) and species of Diapheromeridae clustered into the clade of Phasmatidae. However, some studies support a sister clade of Agathemera and Pseudophasmatidae [5,28].

At the subfamily level, both Lonchodinae and Clitumninae were recovered as a polyphyletic group. In our analysis, we showed that Lonchodinae was not monophyletic because *Phraortes* sp. YW-2014 (Lonchodinae) formed a clade with *Medauroidea extradentata* (Clitumninae) instead of the main clade of Lonchodinae, consistent with previous analyses using molecular markers [115,122] and mitochondrial genomes [6,33,91]. *Phraortes* sp. YW-2014 did not cluster with other *Phraortes* species, probably because of a species misidentification [33]. Meanwhile, some studies, even including transcriptomes, clearly recover the monophyly of Lonchodinae [5,26,28,114]. Therefore, the problem of the monophyly of Lonchodinae (or not) needs further study. *Ph. guangxiensis* (Phasmatidae: Clitumninae) formed a sister group to *Pharnaciini* sp. NS-2020, but the placement of this group and *Phobaeticus serratipes* was distant from the main clade of Clitumninae. In our work, we failed to recover the monophyly of Clitumninae, similar to the results presented in Bradler et al., Robertson et al., and Song et al. [5,22,26].

Analyzing the phylogenetic relationships using ML and BI using the 85 species, Embioptera and Zoraptera were clustered into Phasmatodea, and the monophyly of Phasmatodea was not recovered, which was caused by long-branch attraction. After removal of the Embioptera and Zoraptera species, the phylogenetic relationships of ML and BI using the 81 species showed the monophyly of Phasmatodea and the relationships within subfamilies of Phasmatodea were supported.

## 4. Conclusions

In this study, we successfully determined the complete mitochondrial genomes of *O. guangxiensis*, *Pe. schultei,* and *Ph. guangxiensis.* The three stick insects shared a similar gene arrangement that has been previously reported for other stick insect species. In this study, after removing representatives of Embioptera and Zoraptera that showed long-branch attraction, BI tree and ML trees showed identical topology, except for the position of *Tectarchus ovobessus* (Phasmatodea). We recovered the monophyly of Phasmatodea and showed the sister-group relationship between Phasmatodea and Mantophasmatodea. We recovered the monophyly of Heteropterygidae and the paraphyly of Diapheromeridae, Phasmatidae, Lonchodidae, Lonchodinae, and Clitumninae. In this study, *Peruphasma schultei* (Pseudophasmatidae), *Phraortes* sp. YW-2014 (Lonchodidae), and species of Diapheromeridae clustered into the clade of Phasmatidae. Within Heteropterygidae, *O. guangxiensis* was the sister clade to *O. mouhotii* belonging to Dataminae, and Heteropteryginae was supported as the sister clade to (Dataminae + Obriminae). Future work may need to further explore the mitochondrial genomes of Embioptera and Zoraptera to evaluate the long-branch attraction and explore the phylogenetic relationships between Embioptera and Phasmatodea.

## Figures and Tables

**Table 1:** Species of Phasmatodea used to construct the phylogenetic relationships along with GenBank accession numbers.

Family	Subfamily	Species	Length	Accession no.	Reference
Phylliidae	Phylliinae	*Cryptophyllium tibetense*	18,248 bp	KX091862	Directly Submitted [88]
		*Pulchriphyllium giganteum*	13,980 bp	AB477461	[6,88]
		*Cryptophyllium westwoodii*	17,222 bp	MW229063	[88,89]
Timematidae	Timematinae	*Timema californicum*	14,387 bp	DQ241799	[14]
Bacillidae	Bacillinae	*Bacillus rossius*	14,152 bp	GU001956	[15]
		*Bacillus atticus*	14,141 bp	GU001955	[15]
Heteropterygidae	Dataminae	*Orestes mouhotii*	16,223 bp	AB477462	[6]
		*Orestes guangxiensis*	16,869 bp	MW450873	This study
	Heteropteryginae	*Heteropteryx dilatata*	16,618 bp	AB477468	[6]
Phasmatidae	Megacraniinae	*Megacrania alpheus*	17,124 bp	AB477471	[6]
	Extatosomatinae	*Extatosoma tiaratum*	16,537 bp	AB642680	[6]
	Clitumninae	*Entoria nuda*	16,910 bp	AB477459	[6]
		*Phobaeticus serratipes*	16,182 bp	AB477467	[6]
		*Ramulus hainanense*	15,590 bp	FJ156750	Directly Submitted
		*Ramulus mikado*	16,633 bp	AB477463	[6]
		*Phryganistria guangxiensis*	17,005 bp	MW450875	This study
		*Pharnaciini* sp. NS-2020	15,192 bp	MT025193	[22]
	Phasmatinae	*Dryococelus australis*	16,604 bp	AP018522	[90]
Lonchodidae	Lonchodinae	*Phraortes elongatus*	16,456 bp	AB477460	[6]
		*Megalophasma granulatum*	15,275 bp	KY124331	[91]
		*Phraortes* sp. Miyako Island	14,913 bp	AB477465	[6]
		*Phraortes* sp. Iriomote Island	16,867 bp	AB477464	[6]
		*Phraortes* sp. 1 NS-2020	14,207 bp	MT025191	[22]
		*Eurycantha calcarata*	16,280 bp	MW915467	Directly Submitted
	Necrosciinae	*Micadina phluctainoides*	16,507 bp	AB477466	[6]
		*Sipyloidea sipylus*	17,001 bp	AB477470	[6]
		*Calvisia medogensis*	16,107 bp	KY124330	[91]
		*Neohirasea japonica*	15,305 bp	AB477469	[6]
		*Micadina brachyptera*	15,879 bp	MT025192	[22]
Pseudophasmatidae	Pseudophasmatinae	*Peruphasma schultei*	15,896 bp	MW450874	This study

**Table 2:** Species of other insects (excluding species of Phasmatodea) used to construct the phylogenetic relationships along with GenBank accession numbers.

Order	Species	GenBank No.	References
Archaeognatha	*Nesomachilis australica*	AY793551	[55]
	*Pedetontus silvestrii*	EU621793	[56]
	*Trigoniophthalmus alternatus*	EU016193	[57]
Zygentoma	*Tricholepidion gertschi*	AY191994	[58]
	*Atelura formicaria*	EU084035	[59]
	*Thermobia domestica*	AY639935	[60]
Odonata	*Davidius lunatus*	EU591677	Directly Submitted
	*Somatochlora hineana*	MG594801	Directly Submitted
	*Brachythemis contaminata*	KM658172	[61]
Ephemeroptera	*Siphluriscus chinensis*	HQ875717	[62]
	*Isonychia ignota*	HM143892	Directly Submitted
	*Paegniodes cupulatus*	HM004123	Directly Submitted
	*Serratella zapekinae*	MT274130	[63]
	*Parafronurus youi*	EU349015	[64]
	*Caenis* sp. JYZ-2018	MG910499	[65]
	*Epeorus* sp. JZ-2014	KJ493406	Directly Submitted
	*Epeorus* sp. MT-2014	KM244708	[66]
Plecoptera	*Pteronarcys princeps*	AY687866	[67]
	*Acroneuria hainana*	KM199685	[68]
	*Isoperla bilineata*	MF716959	[69]
Orthoptera	*Locusta migratoria*	X80245	[77]
	*Gastrimargus marmoratus*	EU513373	[70]
	*Oedaleus decorus asiaticus*	EU513374	[70]
Grylloblattodea	*Grylloblatta sculleni*	DQ241796	[14]
Mantophasmatodea	*Sclerophasma paresisensis*	DQ241798	[14]
Mantodea	*Paratoxodera polyacantha*	MG049920	Directly Submitted
	*Mantis religiosa*	MN356097	[71]
	*Pliacanthopus bimaculatus*	MT679725	[72]
Embioptera	*Aposthonia japonica*	AB639034	[34]
	*Aposthonia borneensis*	KX091848	Directly Submitted
	*Eosembia* sp. FS-2017	KX091852	Directly Submitted
Blattodea	*Cryptotermes declivis*	MK599465	[73]
	*Coptotermes formosanus*	AB626147	[74]
	*Macrotermes subhyalinus*	JX144937	[75]
Zoraptera	*Zorotypus medoensis*	JQ910991	Directly Submitted
Dermaptera	*Challia fletcheri*	JN651407	[76]
	*Euborellia arcanum*	KX673196	[36]
	*Eudohrnia metallica*	KX091853	[36]

**Table 3:** Base composition of the mitochondrial genomes of the three species.

Region		*O. guangxiensis*				*Pe. schultei*				*Ph. guangxiensis*			
		Length(bp)	A + T (%)	AT Skew	GC Skew	Length(bp)	A + T (%)	AT Skew	GC Skew	Length(bp)	A + T (%)	AT Skew	GC Skew
mito		16,869	75.6	0.19	−0.22	15,896	76.6	0.16	−0.19	17,005	76.8	0.16	−0.19
PCGs	J	11,112	74.2	0.06	−0.16	11,100	75.5	0.03	−0.12	11,121	75.8	0.06	−0.14
	N			−0.38	0.28			−0.35	0.25			−0.37	0.26
rRNAs		2079	78.3	0.25	−0.32	2055	77.7	0.22	−0.30	2124	77.9	0.20	−0.30
A + T-rich region		2238	79.6	0.17	−0.29	1294	82.5	0.13	−0.37	2286	79.1	0.10	−0.17

## Data Availability

Not applicable.

## Supplementary Materials

The following are available online at https://www.mdpi.com/article/10.3390/insects12090779/s1, Figure S1. Inferred secondary structures of the 22 tRNA genes in *O.* *guangxiensis* mitochondrial genome. A: trnI; B: trnQ; C: trnM; D: trnW; E: trnC; F: trnY; G: trnL (UUA); H: trnK; I: trnD; J: trnG; K: trnA; L: trnR; M: trnN; N: trnS (AGC); O: trnE; P: trnF; Q: trnH; R: trnT; S: trnP; T: trnS (UCA); U: trn L (CUA); V: trnV. Figure S2. Inferred secondary structures of the 22 tRNA genes in *Pe. schultei* mitochondrial genome. A: trnI; B: trnQ; C: trnM; D: trnW; E: trnC; F: trnY; G: trnL (UUA); H: trnK; I: trnD; J: trnG; K: trnA; L: trnR; M: trnN; N: trnS (AGC); O: trnE; P: trnF; Q: trnH; R: trnT; S: trnP; T: trnS (UCA); U: trn L (CUA); V: trnV. Figure S3. Inferred secondary structures of the 22 tRNA genes in *Ph. guangxiensis* mitochondrial genome. A: trnI; B: trnQ; C: trnM; D: trnW; E: trnC; F: trnY; G: trnL (UUA); H: trnK; I: trnD; J: trnG; K: trnA; L: trnR; M: trnN; N: trnS (AGC); O: trnE; P: trnF; Q: trnH; R: trnT; S: trnP; T: trnS (UCA); U: trn L (CUA); V: trnV. Figure S4. The possible secondary structure of the tandem repeat in the control regions. (A) the repeat unit (176 bp) in *O. guangxiensis*. (B) the repeat unit (64 bp) in *Pe. schultei*. (C) the repeat unit (106 bp) in *Ph. guangxiensis*. (D) the repeat unit (128 bp) in *Entoria nuda*. (E) the repeat unit (149 bp) in *Ramulus mikado*. (F) the repeat unit (125 bp) in *Ramulus irregulariterdentatus*. (G) the repeat unit (193 bp) in *Phobaeticus serratipes*. (H) the repeat unit (106 bp) in *Micadina phluctainoides*. (I) the repeat unit (70 bp) in *Phraortes elongatus*. (J) the repeat unit (97 bp) in *Phraortes* sp. Iriomote Island. (K) the repeat unit (21 bp) in *Heteropteryx dilatata*. (L) the repeat unit (149 bp) in *Heteropteryx dilatata*. (M) the repeat unit (64 bp) in *Ramulus hainanense*. (N) the repeat unit (50 bp) in *Megacrania alpheus*. (O) the repeat unit (189 bp) in *Extatosoma tiaratum*. (P) the repeat unit (32 bp) in *Eurycantha calcarata.* Figure S5. Phylogenetic relationships of Phasmatodea inferred from ML analysis including Embioptera and Zoraptera. Figure S6. Phylogenetic relationships of Phasmatodea inferred from BI analysis including Embioptera and Zoraptera. Table S1. Specific primers used to amplify the mitochondrial genomes of *O. guangxiensis*, *Pe. schultei* and *Ph. guangxiensis*. Table S2. Species of Phasmatodea used to construct the phylogenetic relationships along with SRA numbers. Table S3. Location of features in the mtDNA of *O. guangxiensis*. Table S4. Location of features in the mtDNA of *Pe. schultei*. Table S5. Location of features in the mtDNA of *Ph. guangxiensis*. Table S6. Mitochondrial genome comparisons of Phasmatodea species. Table S7. The codon numbers and relative synonymous codon usage in mitochondrial protein-coding genes.
