# Supplementary material for: Three Complete Mitochondrial Genomes of Orestes guangxiensis, Peruphasma schultei, and Phryganistria guangxiensis (Insecta: Phasmatodea) and Their Phylogeny
Source: Insects. 2021 Aug 31;12(9):779. doi: 10.3390/insects12090779 (PMC8471129; doi:10.3390/insects12090779)
Supplement: Supplementary file 1 [file insects-12-00779-s001.zip › TableS1.pdf]

Table S1. Specific primers used to amplify the mitogenomes of *O. guangxiensis*, *Pe. schultei* and *Ph. guangxiensis*.

Species	Primer name	Sequence (5'-3')	Product length (bp)
	11ZJC25-J-503	GTATTTCTCCATTTCACT	1,700
	11ZJC25-N-2246	TGTAGTTTGAGCACATCACA	
	11ZJC25-J-3295	TATAAAAGCAGTAGGACAT	4,200
	11ZJC25-N-7500	CATTCTATCACCAATACG	
O. guangxiensis	11ZJC25-J-7310	ATACAGGGGTAGGAGCA	2,800
	11ZJC25-N-11076	CAACCAGAATGATATTTC	
	11ZJC25-J-11023	CCGCAAATCCCCTAGTAA	3,000
	11ZJC25-N-14056	GCACCTTGACCTGAAATATA	
	11ZJC25-J-14065	ATAAGCTGCACCTTGACC	2,500
	11ZJC25-N-387	ACAAACTATTGCATCAATA	
	ZJC5-J-530	ATTTCACCTTTTCATTTTT	1,900
	ZJC5-N-2438	TTCTTATTCACTCTTGGTGG	
	ZJC5-J-3034	GCAAGATAGATCCTCACCTC	4,300
	ZJC5-N-7362	ATCAAGGTGAAAAAGGAAT	
	ZJC5-J-7354	GCTGGTAATCAGGGAGAA	3,700
Pe. schultei	ZJC5-N-11017	AAGAGACCCGGATAATTTTAC	
	ZJC5-J-11022	GCAAACCCTTTAGTAACACC	2,300
	ZJC5-N-13493	GCGAATAGCATATCACATCTAAG	
	ZJC5-J-14032	AATTATAAGCTGCACCTTG	2,000
	ZJC5-N-358	TATCTAAAGAATCCGCTT	
	11ZJC2-J-773	TAATGGGTGGATAATAGCAG	1,550
	11ZJC2-N-2330	CTGATGTAAAGTAGGCTCGT	
	11ZJC2-J-3071	CATCGCCAATAATAGAACAAC	4,300
	11ZJC2-N-7337	CACCCACTCCTGTATCATCTT	
	11ZJC2-J-9079	GAGAAAGCAACAAACAAAAT	1,700
Ph. guangxiensis	11ZJC2-N-10780	TTATCTACAGCGAAACCC	
	11ZJC2-J-13047	AATGATTATGCTACCTTTG	1,000
	11ZJC2-N-13994	TGAAGGTGGATTTGATAGT	
	11ZJC2-J-14320	AATCCTAGTTTACCGCTTAT	3,200
	11ZJC2-N-567	GTTTCTCCTGTATTTCTTGG	
