# Supplementary material for: Three Complete Mitochondrial Genomes of Orestes guangxiensis, Peruphasma schultei, and Phryganistria guangxiensis (Insecta: Phasmatodea) and Their Phylogeny
Source: Insects. 2021 Aug 31;12(9):779. doi: 10.3390/insects12090779 (PMC8471129; doi:10.3390/insects12090779)
Supplement: Supplementary file 1 [file insects-12-00779-s001.zip › TableS2.pdf]

Table S2. Species of Phasmatodea used to construct the phylogenetic relationships along with SRA numbers.

Family	Subfamily	Species	SRA	Reference
Pseudophasmatidae	Pseudophasmatinae	*Peruphasma schultei*	SRR1002984	[33]
Heteropterygidae	Obriminae	*Aretaon asperrimus*	SRR1172270	[33]
Phylliidae	Phylliinae	*Phyllium siccifolium*	ERR392012	[33]
Phasmatidae	Clitumninae	*Medauroidea extradentata*	SRR1172394	[33]
		Ramulus artemis	SRR1172422	[33]
	Phasmatinae	*Clitarchus hookeri*	SRR3080266	[33]
		Acanthoxyla sp.	SRR2089887	[33]
		Argosarchus horridus	SRR2089902	[33]
Lonchodidae	Lonchodinae	*Phraortes* sp. YW-2014	SRR1189755	[33]
	Lonchodinae	*Carausius morosus*	SRR3211828	[33]
Diapheromeridae	Pachymorphinae	*Spinotectarchus acornutus*	SRR2089897	[33]
		Tectarchus salebrosus	SRR2089908	[33]
		Tectarchus ovobessus	SRR2089893	[33]
		Asteliaphasma jucundum	SRR2089913	[33]
		Micrarchus sp.	SRR1054193	[33]
		Micrarchus hystriculeus	SRR1054191	[33]
		Niveaphasma annulatum	SRR2089878	[33]
