# Supplementary material for: Three Complete Mitochondrial Genomes of Orestes guangxiensis, Peruphasma schultei, and Phryganistria guangxiensis (Insecta: Phasmatodea) and Their Phylogeny
Source: Insects. 2021 Aug 31;12(9):779. doi: 10.3390/insects12090779 (PMC8471129; doi:10.3390/insects12090779)
Supplement: Supplementary file 1 [file insects-12-00779-s001.zip › TableS3.pdf]

Table S3. Location of features in the mtDNA of *O. guangxiensis*

Gene	Strand	Position	Length (nuc.)	Anticodon	Start codon	Stop codon	Intergenic nucleotides
$tRNA^{Ile}$	+	1-67	67	ATC			0
$tRNA^{Gln}$	-	65-133	66	CAA			-1
$tRNA^{Met}$	+	133-198	66	ATG			0
ND2	+	199-1206	1008		ATA	TAA	-2
$tRNA^{Trp}$	+	1205-1268	64	TGA			-8
$tRNA^{Cys}$	-	1261-1327	67	TGC			2
$tRNA^{Tyr}$	-	1330-1396	67	TAC			1
COI	+	1398-2936	1539		ATG	TAA	-5
$tRNA^{Leu2(UUA)}$	+	2932-2994	63	TTA			0
COII	+	2995-3664	670		ATA	T	0
$tRNA^{Lys}$	+	3665-3734	70	AAG			-1
$tRNA^{Asp}$	+	3734-3801	68	GAC			1
ATP8	+	3803-3964	162		ATA	TAA	-4
ATP6	+	3961-4635	675		ATA	TAA	-1
COIII	+	4635-5420	786		ATG	TAA	-1
$tRNA^{Gly}$	+	5420-5484	65	GGA			0
ND3	+	5485-5836	352		ATA	T	0
$tRNA^{Ala}$	+	5837-5903	67	GCA			1
$tRNA^{Arg}$	+	5905-5973	69	CGA			0
$tRNA^{Asn}$	+	5974-6039	66	AAC			0
$tRNA^{Ser1}$	+	6040-6107	68	AGC			0
$tRNA^{Glu}$	+	6108-6172	65	GAA			-2
$tRNA^{Phe}$	-	6171-6238	68	TTC			0
ND5	-	6239-7958	1720		ATT	T	3
$tRNA^{His}$	-	7962-8027	66	CAC			-1
ND4	-	8027-9358	1332		ATG	TAA	-7
ND4L	-	9352-9636	285		ATA	TAA	8
$tRNA^{Thr}$	+	9645-9706	62	ACA			0
$tRNA^{Pro}$	-	9707-9771	65	CCA			1
ND6	+	9773-10252	480		ATT	TAA	0
Cyt *b*	+	10252-11385	1134		ATG	TAA	-1
$tRNA^{Ser2}$	+	11385-11449	65	TCA			-2
ND1	-	11448-12419	972		ATA	TAA	-3
$tRNA^{Leu1(CUA)}$	-	12417-12483	67	CTA			0
16S rRNA	-	12484-13774	1291				0
$tRNA^{Val}$	-	13775-13843	69	GTA			0
12S rRNA	-	13844-14631	788				0
CR		14632-16869	2238				
