# Supplementary material for: Three Complete Mitochondrial Genomes of Orestes guangxiensis, Peruphasma schultei, and Phryganistria guangxiensis (Insecta: Phasmatodea) and Their Phylogeny
Source: Insects. 2021 Aug 31;12(9):779. doi: 10.3390/insects12090779 (PMC8471129; doi:10.3390/insects12090779)
Supplement: Supplementary file 1 [file insects-12-00779-s001.zip › TableS4.pdf]

Table S4. Location of features in the mtDNA of *Pe. schultei*

Gene	Strand	Position	Length (nuc.)	Anticodon	Start codon	Stop codon	Intergenic nucleotides
$tRNA^{Ile}$	+	1-60	60	ATC			13
$tRNA^{Gln}$	-	74-133	60	CAA			0
$tRNA^{Met}$	+	134-203	70	ATG			0
ND2	+	204-1223	1020		ATT	TAA	0
$tRNA^{Trp}$	+	1222-1286	65	TGA			-8
$tRNA^{Cys}$	-	1279-1343	65	TGC			0
$tRNA^{Tyr}$	-	1344-1408	65	TAC			0
COI	+	1409-2947	1539		ATG	TAA	-5
$tRNA^{Leu2(UUA)}$	+	2943-3007	65	TTA			0
COII	+	3008-3677	670		ATT	T	0
$tRNA^{Lys}$	+	3678-3746	69	AAG			0
$tRNA^{Asp}$	+	3747-3811	65	GAC			0
ATP8	+	3812-3970	159		ATC	TAA	-4
ATP6	+	3967-4641	675		ATA	TAA	-1
COIII	+	4641-5429	789		ATG	TAA	-1
$tRNA^{Gly}$	+	5429-5496	68	GGA			0
ND3	+	5497-5848	352		ATA	T	0
$tRNA^{Ala}$	+	5849-5912	64	GCA			0
$tRNA^{Arg}$	+	5913-5978	66	CGA			2
$tRNA^{Asn}$	+	5981-6044	64	AAC			0
$tRNA^{Ser1}$	+	6045-6112	68	AGC			0
$tRNA^{Glu}$	+	6113-6176	64	GAA			-2
$tRNA^{Phe}$	-	6175-6239	65	TTC			0
ND5	-	6240-7962	1723		ATT	T	0
$tRNA^{His}$	-	7963-8025	63	CAC			-1
ND4	-	8025-9356	1332		ATG	TAA	-2
ND4L	-	9355-9640	286		ATG	TAA	2
$tRNA^{Thr}$	+	9643-9705	63	ACA			0
$tRNA^{Pro}$	-	9706-9767	62	CCA			1
ND6	+	9769-10245	477		ATT	TAA	5
Cyt *b*	+	10251-11384	1134		ATA	TAG	-2
$tRNA^{Ser2}$	+	11383-11448	66	TCA			-2
ND1	-	11447-12394	948		ATT	TAA	18
$tRNA^{Leu1(CUA)}$	-	12413-12479	67	CTA			0
16S rRNA	-	12480-13760	1281				0
$tRNA^{Val}$	-	13761-13828	68	GTA			0
12S rRNA	-	13829-14602	774				0
CR	+	14603-15896	1294				
