# Supplementary material for: Three Complete Mitochondrial Genomes of Orestes guangxiensis, Peruphasma schultei, and Phryganistria guangxiensis (Insecta: Phasmatodea) and Their Phylogeny
Source: Insects. 2021 Aug 31;12(9):779. doi: 10.3390/insects12090779 (PMC8471129; doi:10.3390/insects12090779)
Supplement: Supplementary file 1 [file insects-12-00779-s001.zip › TableS5.pdf]

Table S5. Location of features in the mtDNA of *Ph. guangxiensis*

Gene	Strand	Position	Length (nuc.)	Anticodon	Start codon	Stop codon	Intergenic nucleotides
$tRNA^{Ile}$	+	1-69	69	ATC			-3
$tRNA^{Gln}$	-	67-135	69	CAA			-1
$tRNA^{Met}$	+	135-200	66	ATG			-1
ND2	+	201-1219	1019		ATT	TA	0
$tRNA^{Trp}$	+	1219-1288	70	TGA			-8
$tRNA^{Cys}$	-	1281-1344	64	TGC			6
$tRNA^{Tyr}$	-	1351-1418	68	TAC			1
COI	+	1420-2958	1539		ATG	TAA	-5
$tRNA^{Leu2(UUA)}$	+	2954-3020	67	TTA			0
COII	+	3021-3690	670		ATA	T	0
$tRNA^{Lys}$	+	3691-3762	72	AAG			-1
$tRNA^{Asp}$	+	3762-3827	66	GAC			0
ATP8	+	3828-3986	159		ATT	TAA	-4
ATP6	+	3983-4657	675		ATA	TAA	-1
COIII	+	4657-5442	783		ATG	TAA	2
$tRNA^{Gly}$	+	5445-5506	62	GGA			0
ND3	+	5507-5860	354		ATT	TAG	1
$tRNA^{Ala}$	+	5862-5923	62	GCA			2
$tRNA^{Arg}$	+	5926-5990	65	CGA			2
$tRNA^{Asn}$	+	5993-6056	64	AAC			3
$tRNA^{Ser1}$	+	6060-6124	65	AGC			3
$tRNA^{Glu}$	+	6128-6189	62	GAA			4
$tRNA^{Phe}$	-	6194-6259	66	TTC			-1
ND5	-	6259-7983	1725		ATT	TAG	0
$tRNA^{His}$	-	7984-8049	66	CAC			1
ND4	-	8051-9382	1332		ATG	TAG	-7
ND4L	-	9376-9660	285		ATA	TAA	7
$tRNA^{Thr}$	+	9668-9730	63	ACA			0
$tRNA^{Pro}$	-	9731-9801	71	CCA			8
ND6	+	9810-10283	474		ATA	TAA	-1
Cyt *b*	+	10283-11416	1137		ATG	TAA	-1
$tRNA^{Ser2}$	+	11416-11483	68	TCA			-4
ND1	-	11480-12449	970		ATA	T	3
$tRNA^{Leu1(CUA)}$	-	12453-12527	75	CTA			0
16S rRNA	-	12528-13855	1328				0
$tRNA^{Val}$	-	13856-13923	68	GTA			0
12S rRNA	-	13924-14719	806				0
CR		14720-17005	2286				
