# Supplementary material for: Three Complete Mitochondrial Genomes of Orestes guangxiensis, Peruphasma schultei, and Phryganistria guangxiensis (Insecta: Phasmatodea) and Their Phylogeny
Source: Insects. 2021 Aug 31;12(9):779. doi: 10.3390/insects12090779 (PMC8471129; doi:10.3390/insects12090779)
Supplement: Supplementary file 1 [file insects-12-00779-s001.zip › TableS6.pdf]

Table S6. Mitochondrial genome comparisons of Phasmatodea species.

Family	Subfamily	*Species*	Full length (bp)	A (%) (+)	T (%) (+)	C (%) (+)	G (%) (+)	A+T (%) (+)	G+C (%) (+)	AT-skew (+)	GC-skew (+)
Pseudophasmatidae	Pseudophasmatinae	*Peruphasma schultei*	15,896	44.3	32.3	13.9	9.5	76.6	23.4	0.16	-0.19
Timematidae	Timematinae	*Timema californicum*	14,387	38.9	33.2	16.4	11.4	72.1	27.8	0.08	-0.18
Phylliidae	Phylliinae	*Cryptophyllium tibetense*	18,248	48	28	14.2	9.8	76	24	0.26	-0.18
Phylliidae	Phylliinae	*Pulchriphyllium giganteum*	13,980	47	27.6	15.5	9.9	74.6	25.4	0.26	-0.22
Phylliidae	Phylliinae	*Cryptophyllium westwoodii*	17,222	49.2	28.4	13.4	9	77.6	22.4	0.27	-0.19
Bacillidae	Bacillinae	*Bacillus rossius*	14,152	45.9	31.7	13	9.4	77.6	22.4	0.18	-0.16
Bacillidae	Bacillinae	*Bacillus atticus*	14,141	45.9	32.2	12.9	9.1	78.1	22	0.18	-0.17
Heteropterygidae	Dataminae	*Orestes mouhotii*	16,223	44.6	29.7	16.1	9.6	74.3	25.7	0.20	-0.25
Heteropterygidae	Heteropteryginae	*Heteropteryx dilatata*	16,618	45.1	31	14.4	9.5	76.1	23.9	0.19	-0.21
Heteropterygidae	Dataminae	*Orestes guangxiensis*	16,869	44.9	30.7	14.8	9.5	75.6	24.3	0.19	-0.22
Lonchodidae	Lonchodinae	*Phraortes elongatus*	16,456	45.7	31.9	13.1	9.3	77.6	22.4	0.18	-0.17
Lonchodidae	Necrosciinae	*Micadina phluctainoides*	16,507	45.7	32.7	11.8	9.7	78.4	21.5	0.17	-0.10
Lonchodidae	Necrosciinae	*Sipyloidea sipylus*	17,001	45.6	31.7	13.4	9.3	77.3	22.7	0.18	-0.18
Lonchodidae	Lonchodinae	*Megalophasma granulatum*	15,275	43.8	31	14.9	9.7	74.8	24.6	0.17	-0.21
Lonchodidae	Lonchodinae	*Phraortes* sp. Miyako Island	14,913	45.8	31.8	13.2	9.2	77.6	22.4	0.18	-0.18
Lonchodidae	Lonchodinae	*Eurycantha calcarata*	16,280	45	33.2	13.3	8.5	782	21.8	0.15	-0.22
Lonchodidae	Necrosciinae	*Calvisia medogensis*	16,107	44.9	31.8	13.5	9.2	76.7	22.7	0.17	-0.19
Lonchodidae	Necrosciinae	*Neohirasea japonica*	15,305	45.8	33.6	12	8.5	79.4	20.5	0.15	-0.17
Lonchodidae	Lonchodinae	*Phraortes* sp. Iriomote Island	16,867	46.6	32.2	12.1	9.1	78.8	21.2	0.18	-0.14
Lonchodidae	Lonchodinae	*Phraortes* sp. 1 NS-2020	14,207	44.9	30.8	13.3	9.3	75.7	22.6	0.19	-0.18
Lonchodidae	Necrosciinae	*Micadina brachyptera*	15,879	45.1	31.1	13.8	9.7	76.2	23.5	0.18	-0.17
Phasmatidae	Clitumninae	*Pharnaciini* sp. NS-2020	15,192	46.1	31.9	12.7	9.2	78	21.9	0.18	-0.16
Phasmatidae	Clitumninae	*Phryganistria guangxiensis*	17,005	44.6	32.2	13.8	9.4	76.8	23.2	0.16	-0.19
Phasmatidae	Megacraniinae	*Megacrania alpheus*	17,124	46.2	30.6	13.9	9.3	76.8	23.2	0.20	-0.20
Phasmatidae	Clitumninae	*Entoria nuda*	16,910	43.8	32.2	13.8	10.2	76	24	0.15	-0.15
Phasmatidae	Clitumninae	*Phobaeticus serratipes*	16,182	45.9	31.1	14	9	77	23	0.19	-0.22
Phasmatidae	Clitumninae	*Ramulus hainanense*	15,590	43	30.1	16.3	10.6	73.1	26.9	0.18	-0.22

Phasmatidae	Clitumninae	*Ramulus mikado*	16,633	43.2	32.8	13.3	10.7	76	24	0.14	-0.11
Phasmatidae	Extatosomatinae	*Extatosoma tiaratum*	16,537	45.6	30.3	15.1	8.9	75.9	24	0.20	-0.26
Phasmatidae	Phasmatinae	*Dryococelus australis*	16,604	44.8	32.6	13	9.5	77.4	22.5	0.16	-0.6
