# Supplementary material for: Three Complete Mitochondrial Genomes of Orestes guangxiensis, Peruphasma schultei, and Phryganistria guangxiensis (Insecta: Phasmatodea) and Their Phylogeny
Source: Insects. 2021 Aug 31;12(9):779. doi: 10.3390/insects12090779 (PMC8471129; doi:10.3390/insects12090779)
Supplement: Supplementary file 1 [file insects-12-00779-s001.zip › TableS7.pdf]

Table S7. Codon numbers and relative synonymous codon usage in mitochondrial protein coding genes of *O. guangxiensis* (OG), *Pe. schultei* (PS) and *Ph. guangxiensis* (PG).

**Codon**	**Count**			**RSCU**			**Codon**	**Count**			**RSCU**		
	OG	PS	PG	OG	PS	PG		OG	PS	PG	OG	PS	PG
**UUU(F)**	284	293	288	1.62	1.72	1.69	GCG(A)	5	3	2	0.16	0.1	0.07
**UUC(F)**	66	48	52	0.38	0.28	0.31	UAU(Y)	143	161	163	1.68	1.74	1.86
**UUA(L)**	325	345	354	3.98	4.11	4.26	UAC(Y)	27	24	12	0.32	0.26	0.14
**UUG(L)**	59	58	53	0.72	0.69	0.64	CAU(H)	49	56	56	1.44	1.75	1.65
**CUU(L)**	34	42	42	0.42	0.5	0.51	CAC(H)	19	8	12	0.56	0.25	0.35
**CUC(L)**	4	3	1	0.05	0.04	0.01	CAA(Q)	59	56	61	1.84	1.81	1.91
**CUA(L)**	60	43	46	0.73	0.51	0.55	CAG(Q)	5	6	3	0.16	0.19	0.09
**CUG(L)**	8	13	3	0.1	0.15	0.04	AAU(N)	150	149	158	1.56	1.54	1.62
**AUU(I)**	279	333	334	1.63	1.79	1.74	AAC(N)	42	45	37	0.44	0.46	0.38
**AUC(I)**	64	39	51	0.37	0.21	0.26	AAA(K)	83	89	89	1.66	1.68	1.66
**AUA(M)**	343	299	328	1.83	1.81	1.83	AAG(K)	17	17	18	0.34	0.32	0.34
**AUG(M)**	31	32	31	0.17	0.19	0.17	GAU(D)	60	59	56	1.69	1.74	1.62
**GUU(V)**	84	81	83	1.73	1.81	1.94	GAC(D)	11	9	13	0.31	0.26	0.38
**GUC(V)**	6	5	5	0.12	0.11	0.12	GAA(E)	62	69	61	1.65	1.73	1.54
**GUA(V)**	87	84	77	1.79	1.88	1.8	GAG(E)	13	11	18	0.35	0.28	0.46
**GUG(V)**	17	9	6	0.35	0.2	0.14	UGU(C)	42	40	43	1.83	1.78	1.83
**UCU(S)**	100	104	98	2.37	2.39	2.43	UGC(C)	4	5	4	0.17	0.22	0.17
**UCC(S)**	12	15	4	0.28	0.34	0.1	UGA(W)	72	86	84	1.52	1.77	1.79
**UCA(S)**	100	107	102	2.37	2.46	2.53	UGG(W)	23	11	10	0.48	0.23	0.21
**UCG(S)**	5	7	3	0.12	0.16	0.07	CGU(R)	16	14	23	1.21	1.08	1.74
**CCU(P)**	52	69	58	1.54	2.11	1.69	CGC(R)	2	4	0	0.15	0.31	0
**CCC(P)**	14	5	17	0.41	0.15	0.5	CGA(R)	25	34	28	1.89	2.62	2.11
**CCA(P)**	61	48	58	1.81	1.47	1.69	CGG(R)	10	0	2	0.75	0	0.15
**CCG(P)**	8	9	4	0.24	0.27	0.12	AGU(S)	34	35	37	0.81	0.8	0.92
**ACU(T)**	76	71	63	1.49	1.36	1.21	AGC(S)	7	5	10	0.17	0.11	0.25
**ACC(T)**	26	19	24	0.51	0.36	0.46	AGA(S)	74	73	63	1.76	1.68	1.56
**ACA(T)**	99	118	116	1.94	2.26	2.22	AGG(S)	5	2	6	0.12	0.05	0.15
**ACG(T)**	3	1	6	0.06	0.02	0.11	GGU(G)	105	96	102	2.06	1.89	2
**GCU(A)**	59	54	54	1.83	1.8	1.83	GGC(G)	10	6	3	0.2	0.12	0.06
**GCC(A)**	16	4	10	0.5	0.13	0.34	GGA(G)	51	74	71	1	1.46	1.39
**GCA(A)**	49	59	52	1.52	1.97	1.76	GGG(G)	38	27	28	0.75	0.53	0.55
